# Yoga and Mindfulness for Successful Aging: An Indigenous Life Course Perspective From South Asia

**DOI:** 10.1093/geroni/igaf122.2958

**Published:** 2025-12-31

**Authors:** Kamala Ramadoss, Ishan Shivanand

**Affiliations:** Syracuse University, Syracuse, New York, United States; Yoga of Immortals (USA) and Indian Institute of Technology (India), Manlius, New York, United States

## Abstract

Research on yoga and mindfulness-based interventions have focused on the positive impact of yoga and mindfulness-based interventions on health, wellbeing and successful aging. However, some researchers have simultaneously cautioned about weak effects and/or lack of evidence about the effectiveness of yoga and mindfulness-based interventions and the decline in the adoption of yoga and mindfulness as alternative therapies. A major gap in the literature is a lack of critique about the basic concepts and theoretical considerations, as the variables used to measure these concepts need to be grounded in theoretical rigor. In this paper, the authors will examine the history and key concepts of yoga and mindfulness from an indigenous perspective from the Indian sub-continent where these methods originated, along with a discussion on how it contributes to successful aging. Specifically, yoga of immortals from a life course perspective and the concept of “ichha mrutyu” (living a full and healthy life and leaving the body when one has finished living a full and healthy life in a manner and at a time of one’s own choosing – not to be confused with euthanasia) versus death (where one has no control) will be discussed. Simultaneously, the authors will examine how these concepts have been adopted in the West and the gaps that arise from this translation of these conceptualizations from East to West. Consequently, the validity and vigor of research, teaching and practice of yoga and mindfulness-based interventions for successful aging will be strengthened.

